# Perioperative Risk Stratification with AI-Powered Chatbots: A Systematic Review and Meta-Analysis

**DOI:** 10.3390/jcm15124670

**Published:** 2026-06-16

**Authors:** Valentina Bellini, Matteo Panizzi, Stefano Delrio, Michele Berdini, Victor Sapountzakis, Luis Antonio dos Santos Diego, Elena Giovanna Bignami

**Affiliations:** 1 Anesthesiology, Critical Care and Pain Medicine Division, Department of Medicine and Surgery, University of Parma, Viale Gramsci 14, 43126 Parma, Italy; valentina.bellini@unipr.it (V.B.); matteo.panizzi@unipr.it (M.P.); stefano.delrio@unipr.it (S.D.); michele.berdini@unipr.it (M.B.); 2 Anesthesiology and Perioperative Medicine Division, Servicos Medicos de Anestesia (SMA), Rua Conselheiro Brotero 1486, São Paulo 01232-910, Brazil; victorsapountzakis@gmail.com; 3 Departamento de Anestesiologia, Universidade Federal Fluminense (UFF), Rio de Janeiro 24070-090, Brazil; luisdiego@id.uff.br

**Keywords:** artificial intelligence, chatbot, perioperative, risk management, risk assessment, anesthesia planning, risk stratification, clinical decision support

## Abstract

**Background**: Chatbots are becoming increasingly valuable in clinical settings, offering rapid access to medical information, aiding documentation, and improving perioperative patient education. Their adaptability makes them promising tools for personalized perioperative risk stratification (PRS) and anesthesia planning, but their definitive role remains uncertain. We aimed to evaluate chatbot performance in PRS compared to standard clinical judgment and to assess the certainty of the evidence supporting their use. **Methods**: This systematic review (PROSPERO ID: CRD42025642357) followed PRISMA extended and PRISMA-S guidelines. The population was defined according to the PICO framework: we included adult surgical patients undergoing anesthesia assessment (P), evaluated with LLM-based chatbots for perioperative risk stratification and anesthesia planning (I), compared with traditional clinician assessment (C), and extracted performance metrics (O). Comprehensive searches of PubMed, MEDLINE, Scopus, Embase, Google Scholar, Open Gray, ClinicalTrials.gov, WHO ICTRP, and Cochrane Library Central were conducted through January 2026. Risk of bias and study quality were assessed using PROBAST-AI, RoB-2, and ROBINS-I. Certainty of the evidence was assessed using GRADE system. A random-effects meta-analysis of pooled chatbot accuracy was performed, with subgroup analyses by ASA status and perioperative risk stratification. A sensitivity analysis was performed with a leave-one-out exclusion test. **Results**: Eleven studies published between 2023 and January 2026 were included (N = 227,059 patients). Five prospective cohorts, two large retrospective cohorts, one randomized non-inferiority trial, and three non-clinical or mixed-methods studies were found. Meta-analysis showed that the pooled accuracy of LLM-based chatbots for AI–clinician concordance in perioperative risk stratification and ASA classification was 0.90 [95% CI: 0.42–0.99; 95% prediction interval 0.03–1.00]. Subgroup analyses indicated that the ASA status prediction subgroup reached a pooled accuracy of 0.91 (95% CI: 0.46 to 0.99), whereas the exploratory perioperative risk stratification subgroup showed an accuracy of 0.73 (95% CI: 0.10 to 0.98). Performance decreased with increasing patient complexity. Evidence is limited by small sample sizes, extreme sample size skew toward a single center, geographic bias, inconsistent outcome definitions and performance metrics, and incomplete reporting of adverse events. Most studies lacked prospective trial registration or robust control for confounding, and publication bias cannot be excluded. **Conclusions**: LLM-based chatbots show promising performance in routine perioperative risk stratification but remain unreliable in complex cases, with potential safety concerns. Given the overall very low GRADE certainty of evidence, these tools should be used as clinician-supervised decision support aids for routine ASA assessment, and should not be relied upon for autonomous use in complex cases or for general perioperative risk stratification. **Other:** This research received no external funding. PROSPERO ID: CRD42025642357.

## 1. Introduction

Natural language processing (NLP) is a branch of artificial intelligence (AI) that uses machine learning (ML) to make computers communicate through human language. Large language models (LLMs) are advanced NLP pre-trained language models (PLMs) and are the engine for chatbots. Chatbots are AI-powered software designed to simulate and process human conversation (written or spoken), permitting users to interact with digital devices as if they were communicating with a real person. There are two main categories: rule-based chatbots, which follow pre-defined scripts, and AI-based chatbots, which use ML and NLP to understand and respond dynamically [1]. ML-based chatbots are becoming increasingly popular and are employed for different medical purposes: patients increasingly use them to seek answers to medical queries, and physicians are beginning to use these tools as clinical decision support systems (CDSs) [2,3,4]. Currently, their primary use is focused on patient education and preoperative preparation, aiming to empower patients and enhance compliance to ensure optimal conditions before the procedure. In the postoperative phase, they are utilized for pain monitoring and management, as well as for promoting rapid recovery after surgery through bidirectional feedback mechanisms [5,6,7]. In this field, the most popular chatbots are ChatGPT (OpenAI), Bard (Google), and Bing Chat (Microsoft). ChatGPT, the most widely used, provides quick access to information on medical topics, assisting in generating medical and scientific papers, performing medical data analyses, and even interpreting complex datasets [8,9]. Various predictive approaches have been tested to automatically classify preoperative patient risk, such as the American Society of Anesthesiologists Physical Status (ASA), to detect respiratory depression during conscious sedation and to assist in decision-making regarding the optimal anesthesia method for surgery, even if they often lack external validation [10,11,12]. Traditionally, perioperative evaluation has been conducted by healthcare professionals through patient interviews, medical history reviews, physical examinations, and laboratory tests, but this process is time-consuming, resource-intensive, repetitive, and prone to human error. AI models trained on patient characteristics and outcomes from previous surgeries could potentially predict, with high accuracy, the outcomes from multiple anesthetic and surgical options [13,14]. Chatbots may support many of the tasks mentioned above, making them a valuable tool in risk management and patient care optimization by streamlining preoperative data collection, reducing surgical complications associated with incomplete or insufficient information and enabling patients to better understand the risks and benefits of their procedures by drawing a comprehensive picture of the patient’s health status [15].

### Objectives

The quality of the interaction between clinicians and AI is fundamental for acceptance and for the effective integration of LLMs into clinical workflows: the perceived quality of interaction between clinicians and AI is closely linked to user experience, and clinicians with a positive perception are more likely to adopt AI systems [16,17]. However, their role in enhancing preoperative risk evaluation remains undefined and there is an urgent need for systematic, high-quality evidence on when and how chatbots can safely support perioperative evaluation. Shih-Jung Lin and colleagues have already conducted a systematic review of the perioperative use of chatbots from various perspectives, but their role in risk stratification has not been specifically evaluated [14]. In this systematic review, we examine the current applications of LLM-based chatbots in preoperative patient risk assessment with the aim of establishing certainty of the evidence supporting their use in everyday practice.

## 2. Materials and Methods

### 2.1. Eligibility Criteria

This systematic review was conducted according to the Extended Preferred Reporting Items for Systematic Reviews and Meta-Analyses (PRISMA) 2020 statement and PRISMA-S [Supplementary Materials, Tables S1 and S2] [18,19]. The search population was defined according to the PICO framework [Table 1].

Population

Adult patients (≥18 years of age) undergoing perioperative assessment in any clinical setting (inpatient, outpatient, or emergency departments) and any surgical specialty.

Intervention

The intervention of interest was the use of large language model-based chatbots used for perioperative patient risk assessment. Both proprietary models and open-source or freely available models were eligible.

Comparator

The reference standard was assessment by human expert clinicians. Studies comparing AI outputs to gold-standard ASA classifications determined by experienced clinicians, consensus panels, or validated reference standards were eligible.

Outcomes

The performance of LLM-based chatbot systems in perioperative risk stratification compared to standard clinical judgment was assessed in terms of the area under the receiver operating characteristic (AUROC), accuracy, specificity, sensitivity and agreement with clinician’s opinion. The anesthetic technique chosen was considered part of risk stratification because of its correlation with patients’ health status.

Inclusion criteria: (1) papers studying adult patients undergoing surgical procedures requiring anesthesia and perioperative anesthesiologic assessment, and (2) perioperative risk stratification or anesthesia planning was performed with chatbots powered by large language models (LLMs).

Exclusion criteria: (1) study protocols, correspondence and reviews; (2) non-surgical treatment or routine procedures without anesthesiologic assessment; (3) papers that do not study risk stratification or anesthesia planning; (4) studies focusing on the education of patients or clinicians or on clinician–patient communication; (5) papers evaluating non-chatbot large language models (LLMs) with no direct perioperative risk stratification or anesthesia decision support application (e.g., generic NLP pipelines or purely technical model benchmarks).

In addition, models for perioperative risk prediction that were not frontend chatbots were retained as complementary evidence on LLM-based perioperative decision support tools and were not pooled with chatbot studies in the main meta-analysis.

Two reviewers screened independently titles and abstracts. Articles meeting inclusion criteria were retrieved in full text for assessment. Full-text screening was performed by the same two independent reviewers using a standardized data abstraction form. In case of discordance, the two examiners reviewed disagreements jointly and reached consensus through discussion until reaching 100% agreement.

The review protocol was defined by all authors before the search and registered on PROSPERO in January 2025 (ID: CRD42025642357), amended in November 2025 due to the extension of the search strategy, and in May 2026 at the end of the review to improve the clarity of the PICO framework without changing the core research structure.

The authors declare no conflicts of interest, including financial or personal relationships with organizations that could inappropriately influence this work. They have no relationships with companies developing or commercializing the chatbots or AI systems discussed in this manuscript, and no funding was received.

### 2.2. Information Sources and Search Strategy

We conducted a comprehensive search of the electronic databases of PubMed, MEDLINE, Scopus, and Embase for peer-reviewed studies, and Google Scholar (Scholar Labs), Open Gray, ClinicalTrials.gov, WHO ICTRP, and Cochrane Library Central for gray literature from their inception to January 2026. The search strategy combined medical subject headings (MeSHs) and keywords organized into three concept groups: (1) artificial intelligence terminology (chatbot, artificial intelligence, AI, machine learning, ML, deep learning, DL, large language models, LLM, ChatGPT, GPT-4, GPT-*, gemini, copilot *, claude *, deepseek *, natural language processing, Mixtral, LLaMA, NPL, virtual assistant, digital assistant, conversational agent, intelligent virtual agent); (2) clinical application (perioperative, preoperative, preoperative assessment, preoperative evaluation, anesthesia, anesthesia consultation, ASA, ASA physical status, ASA classification, ASA-PS, risk assessment, risk stratification, surgical risk, anesthetic risk, anesthesiologic risk, NSQIP, AKI, AKI risk, Mini-Cog, SORT, p-possum, EuroSCORE-II, frailty, clinical frailty risk); and (3) outcome measures and comparators (accuracy, performance, validation, classification, prediction, clinician assessment, clinician concordance). Boolean operators were used to combine these search terms. The complete search strategy can be found in the Supplementary Materials [File S1]. For PubMed, we implemented a Python (Python Software Foundation, Beaverton, OR, USA, Software version 3.13) script that queried the API using the predefined search string, downloaded all records, and performed automated de-duplication based on DOI, PMID and title before manual screening. The script did not alter the search logic but ensured complete retrieval and systematic deduplication; all unique records were subsequently verified in the PubMed interface.

No published search filters were used, and no search strategies of previous work were reused. No additional studies or data were sought by contacting experts, manufacturers, or others. Two different authors revised the results independently.

### 2.3. Data Extraction and Synthesis

Data from the selected studies were extracted and summarized using an Excel table. Data were independently extracted by two reviewers using a standardized form that captured study characteristics (author, year, country, study design), population demographics (sample size, age, gender, ASA physical status, concordance), AI system specifications, reference standards used, and outcome measures according to Cochrane guidelines [20]. For each study, AI–clinician concordance for categorical outcomes (e.g., ASA-PS) was extracted as accuracy, defined as the proportion of identical classifications between the AI system and the reference standard. Using Cohen’s kappa (κ) to infer accuracy requires specifying the expected chance agreement (p_c_), which depends on the marginal distributions of the raters and can vary substantially between settings. To avoid bias in the presence of imbalance, whenever the underlying contingency table was available, we computed accuracy directly from observed counts rather than from κ. When raw accuracy could not be derived from contingency tables, we approximated accuracy as accuracy ≈ κ (1 − P_c_) + P_c_, where P_c_ represents the expected agreement by chance. When base rates were unavailable, κ was used as a proxy for the observed concordance percentage.

### 2.4. Statistical Analysis

#### 2.4.1. Meta-Analysis of AI–Clinician Concordance Accuracy

Statistical analysis and forest plots were performed using RStudio (Posit Software, PBC, Boston, MA, USA, software version 4.5.2, graphical user interface 1.82). Meta-analysis of proportions was performed using the meta package, with the metaprop() function for pooling proportions and byvar for subgroup analyses (Q statistic). AI–clinician pooled accuracy was synthesized across studies reporting comparable accuracy outcomes. Accuracy was treated as a binomial proportion and analyzed on the logit scale. AI–clinician concordance proportions were logit-transformed and combined using the inverse-variance method. A random-effects meta-analysis was performed on the logit scale, estimating the between-study variance (τ^2^) via restricted maximum likelihood (REML). Pooled estimates and 95% confidence intervals were calculated using the Hartung–Knapp–Sidik–Jonkman method to account for the expected high heterogeneity and the small number of studies included. We additionally report I^2^, τ^2^, and the random-effects prediction interval.

#### 2.4.2. Subgroup Analyses

Random-effects meta-analyses were performed for two subgroups: (1) performance in predicting ASA status and (2) performance in evaluating perioperative risk. Heterogeneity within each subgroup was assessed, and between-group differences were evaluated using the Q-between statistic to test for significant subgroup effects.

#### 2.4.3. Non-Poolable Outcomes Analysis

AUROC for complication prediction, odds ratios for complication identification improvement, decision quality scores, and clinical utility measures were not meta-analyzed due to heterogeneous outcome constructs, different measurement scales, and insufficient number of studies (most reported by single studies only). These outcomes are presented descriptively.

### 2.5. Risk of Bias and Quality Assessment

Risk of bias (RoB) was assessed using PROBAST-AI for prediction model studies, RoB-2 for randomized clinical trials, and ROBINS-I for non-randomized comparative/observational studies. Each domain was rated as low, moderate, high and serious risk of bias.

Certainty of evidence for each outcome was assessed using the Grading of Recommendations, Assessment, Development and Evaluation (GRADE) approach, systematically evaluating risk of bias, inconsistency, indirectness, imprecision and publication bias [21].

Because of methodological and outcome heterogeneity among the included studies, the GRADE system was used thanks to its known appropriateness in qualitative synthesis even if a meta-analysis could not be performed for some outcomes [21,22].

## 3. Results

### 3.1. Study Selection Process

**Figure 1:**
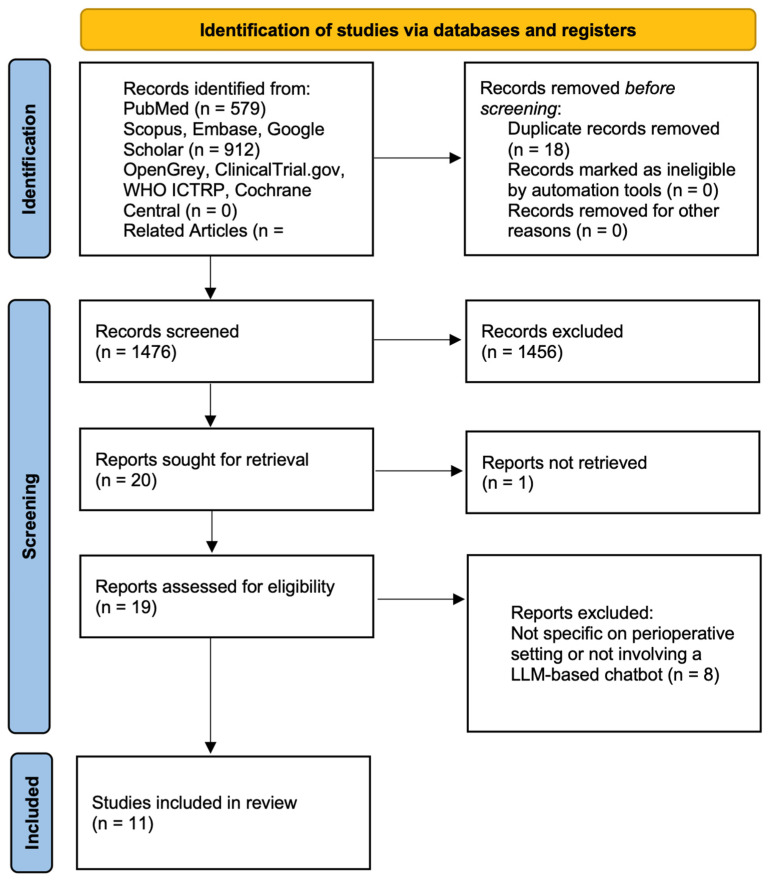
A flow diagram of the study selection process according to PRISMA guidelines, showing identification, screening, eligibility assessment, and inclusion of studies from PubMed, MEDLINE, Scopus, Embase, Google Scholar, Open Gray, ClinicalTrials.gov, WHO ICTRP, and Cochrane Library Central from inception to January 2026. No papers were found in Open Gray or WHO ICTRP. The same search strategy was used for each database mentioned.

A total of 1494 papers were found. After the removal of duplicates, 1476 were screened and 1456 were then excluded. Twenty records were sought for retrieval: one record full text was retrieved by contacting the corresponding author and one was not retrieved because no full text was available through databases or journal website. Then, 19 papers were assessed for eligibility. Eight papers were excluded after full-text review because they were not specific to the perioperative period or did not involve an LLM-based chatbot [Figure 1]. One registered trial was still ongoing. No papers were found in Open Gray or WHO ICTRP.

### 3.2. Study Characteristics and Sample

Eleven studies published between 2023 and January 2026 were included. The combined sample reached N = 227,059. Large variation in study sample size was observed ranging from 10 to 137,535 patients per study. Study designs also varied considerably: we found two retrospectives (N = 137,535 and N = 84,875), five prospectives (N = 3594), one non-inferiority randomized controlled trial (N = 600), one comparative, non-clinical (N = 295), one case–control (N = 150) and one mixed method (N = 10). As shown in Table 2, two papers account for 97.9% of total patients.

The mean participant age ranged from 45 to 58 years across studies, with the sex distribution generally balanced (46% to 55% male). ASA distribution across studies was as follows: 34.5% ASA I-II patients, 46.2% ASA III patients, and 19.2% ASA IV-V patients. Surgical specialties represented included general surgery (38%), orthopedic surgery (22%), gynecologic surgery (15%), urologic surgery (12%), and mixed/other specialties (13%) [Table 3].

Most investigations originated from Asia (seven studies, N = 4238, 1.8% of total patients), whereas North America alone accounts for 97.9% of all patients [Table 4].

### 3.3. Risk of Bias and Study Quality Assessment

Overall, domain-based assessment indicated a predominantly high-risk evidence base, with 6/11 studies rated serious, 3/11 moderate–high, 1/11 moderate, and 1/11 low–moderate RoB. The most frequent limitations were selection bias because of restricted populations or simulated/proxy designs, the presence of confounding in non-randomized comparisons, and outcome/reporting limitations when reference standards were subjective or poorly standardized across settings. For prediction model studies using real-world clinical data, PROBAST-AI highlighted concerns mainly in analysis and outcome domains (rare events, missing outcome assessment, limited validation details), supporting a conservative interpretation of model performance and clinical usability. Eight of ten studies (80%) lacked registration on ClinicalTrials.gov or equivalent registries prior to patient enrollment. The unique study with prospective registration was counterbalanced by another investigation registered 15 months after enrollment commenced—essentially a retroactive documentation. The remaining eight studies provided no accessible registration records. A summary of RoB assessment is provided in [Table 5].

About half of the studies limited their sample in ways that make it hard to generalize the findings: some focused almost exclusively on unusually complex cases, others deliberately removed difficult airways, comatose patients or cardiac murmurs from consideration, one relied on examination questions instead of real perioperative cases, and another included only ASA III orthopedic and geriatric patients, leaving out the usual mix of lower-risk individuals, overall introducing selection bias. Outcome assessment entailed additional problems. In one study, eleven different postoperative complications were bundled into a single composite endpoint and recorded by telephone six months after surgery, with very few events overall, a combination that makes the results highly sensitive to recall errors and random variation. Another study faced the opposite difficulty: very rare complications such as pulmonary embolism and mortality stood alongside a much more frequent outcome, delirium, for which most screening data were missing; under these conditions, prediction models inevitably tend to favor the common outcome and perform poorly on the rare but clinically important ones.

Confounding was rarely handled in a structured way. In most reports, comparison groups and statistical adjustment were insufficient to separate the effect of the AI tool from underlying differences in case mix, while only two studies used designs with randomization or standardized consecutive inclusion and clearly defined comparators. A final pattern concerns how results are reported: every included study described some degree of benefit associated with AI, and none concluded that the systems were ineffective, a symmetry that is unusual in clinical research and consistent with a publication landscape that favors positive findings.

### 3.4. Descriptive Overview

The most frequent quantitative evaluation methods were AUROC, AUPRC, and accuracy for risk/complication prediction. Models achieved an AUROC of 0.74–0.87, with a lower AUPRC for rare events, very high sensitivity, but low specificity [23,24,26]. Cohen’s kappa or concordance rate was widely used when comparing chatbot outputs to expert assessments for categorical tasks (ASA score prediction, treatment recommendation): the highest Cohen’s kappa was κ = 0.85 and an accuracy higher than 90% was achieved for typical cases and ASA I–III patients (down to 63.9% for complex cases and ASA IV) [11,25,32]. However, for complex anesthesia planning, agreement was substantially lower: Malek et al. (2024; n = 10 simulated cases) reported κ = 0.1–0.4 (poor agreement) for ChatGPT-generated anesthetic plans, with >33% of recommendations (n ≥ 3/10) evaluated as unsafe or non-standard by expert review. This contrasted sharply with routine ASA classification (κ = 0.85) and indicates critical safety concerns for autonomous planning in complex scenarios [32]. Percent agreement with clinician assessments varied by chatbot model and clinical task: Gemini demonstrated 68.5% agreement for spinal anesthesia selection (n = 49/72 patients) and 85.7% for patients taking medications (n = 62/72), while overall clinician–AI concordance ranged from 60 to 70% for typical cases across other models [27,29]. The highest objective scores were found in structured tasks like ASA or risk prediction but chatbots struggle in high-complexity, context-sensitive, or adversarial/edge-case scenarios like open-ended or generative planning tasks or rare event predictions.

Various chatbots were studied including ChatGPT, Gemini, Grok, Deep Seek and Copilot. In the study by Celik et al., the highest accuracy of output in predicting the type of anesthesia and risk stratification, for those patients that take medications, was reached by Gemini (85.7% of cases) [30]. In contrast, Malek et al. showed that responses provided by ChatGPT were repetitive and lacked variety, showing a marked preference for general anesthesia while neglecting regional techniques. Furthermore, the choices regarding airway management, postoperative analgesia, and medication use were inconsistent. The preoperative anesthetic plans generated by ChatGPT did not consistently meet minimum clinical standards and appeared to lack genuine clinical reasoning [32].

The largest RCT to date (Qi et al., n = 600 adult patients undergoing elective non-cardiac surgery) demonstrated non-inferior ASA evaluation accuracy for the AI-assisted group (93.3% [n = 280/300] vs. 91.7% [n = 275/300]; 95% CI −2.6% to 5.9%, *p* = 0.033) versus in-person traditional assessment, 50% superior documentation quality (n = 600 patients, *p* < 0.001), faster evaluation time with a 57% reduction in evaluation time (median 3 min vs. 7 min; *p* < 0.001), and higher patient satisfaction scores (≥95%; *p* < 0.001). [25] The study states that AI-based evaluation systems can enhance the efficiency, quality, and acceptance of preoperative anesthesia assessments without compromising diagnostic accuracy [25].

Ruan et al. evaluated four large language models (ChatGPT-4o, Claude 3.5 Sonnet, DeepSeek-R1, Grok 3) in supporting anesthesia decision-making for 30 complex clinical cases (obstetric and geriatric patients, ASA ≥ III). [31] DeepSeek-R1 achieved the highest overall multidimensional score (51.43/60; *p* < 0.001 compared to other models), outperforming other models in both obstetric cases (mean [SD] 52.00 [1.83]) and geriatric cases (mean [SD] 51.15 [3.10]). However, complex case accuracy decreased to 34.5% in highly complex geriatric scenarios, significantly lower than that of standardized obstetric cases (*p* < 0.001), demonstrating performance degradation in multimorbid patient contexts. The authors concluded that LLMs may serve as valuable initial decision support tools, but clinical supervision remains essential [31].

The papers by Ke et al. (2025 and 2026) evaluated the clinical and economic impact of PEACH (PErioperative AI CHatbot). [28,33] PEACH did not significantly reduce overall documentation time (n = 272 interactions; mean 19.35 [SD 10.95] vs. 17.53 min [SD 11.45]; *p* = 0.19), but improved time efficiency for moderate-complexity cases (−5.77 min, [SD 7.0], n = 101, *p* = 0.01) and experienced physicians (−4.62 min, [SD 7.2], n = 99, *p* = 0.04). Documentation quality improved with higher completeness of clinical issue lists (71.4% [50/70] vs. 43.9% [31/70]; χ^2^ = 4.18, *p* = 0.05; n = 272 interactions) and 57.1% evaluator preference. The system demonstrated high accuracy (96.7%, improved to 97.9% after protocol refinement) and minimal hallucinations (0.4–1.7% over n = 272 interactions) and deviations (0.8% over n = 272 recommendations), with 100% appropriate disclaimers (n = 272/272; 95% CI 99.3–100%). Strong inter-rater reliability (κ = 0.77–0.89 across domains) and 91.4% clinical concordance (53/58 evaluations, 95% CI 81.0–96.9%) with clinicians were observed [28,33].

Specifically, regarding the risk stratification performed with existing validated clinical scoring tools, only the prediction of ASA score was extensively studied [11].

We could not find any other risk stratification tool such as Surgical Outcome Risk Tool (SORT) or cardiac or frailty risk assessment evaluated through a chatbot. However, Alba et al. (2025) compared domain-specific pre-trained LLMs (BioGPT, ClinicalBERT, BioClinicalBERT) in predicting six significant postoperative complications (30-day mortality, pulmonary embolism, deep vein thrombosis, acute kidney injury, delirium, pneumonia) using preoperative clinical notes from 84.875 surgical cases. [24] These biomedical/clinical domain-specific models significantly outperformed traditional NLP models (*p* < 0.001), with AUROC ranging from 0.74 to 0.87 (mean [SD] 0.80 [0.05]) and AUPRC up to 0.67 for rare events, achieving performance comparable to the National Surgical Quality Improvement Program (NSQIP) Surgical Risk Calculator, with equivalent or superior accuracy and F1 scores. In this context, sensitivity was slightly lower (mean difference −8.4% [SD 6.4]; *p* = 0.02), particularly for rare complications. These findings suggest domain-specific models show promise for complication prediction while highlighting current limitations for autonomous clinical deployment [24]. Even if the paper does not mention explicitly the use of a bidirectional chatbot, bidirectional tools such as Large Finetuned Chatdoctor based on BioGPT exist.

Regarding clinicians’ opinion, the performance of ChatGPT’s ASA score prediction was evaluated by the authors as satisfying and it correlates with clinical judgment. According to Celik et al., it serves as a valuable assistant rather than replacing doctors and it can assist anesthesia practitioners and surgeons by alerting them to the ASA-PS classification and assessing perioperative risk [30,32]. ChatGPT-4 is efficient in medical data analysis, especially in ASA score assessing, even if ChatGPT is currently not recommended for preoperative anesthetic planning [11,32]. Study characteristics are summarized below in Table 6.

### 3.5. AI–Clinician Accuracy Meta-Analysis

Four prospective studies (N = 3607) with a homogeneous study design and endpoints contributed data to the primary meta-analysis of AI–clinician accuracy evaluation. Only studies evaluating LLM-based chatbots were entered into the primary meta-analysis. Other non-chatbot LLM implementations were analyzed descriptively only and were excluded from the pooled chatbot accuracy estimates.

In the prospective multicenter study by Turan et al., ChatGPT-4 ASA agreement with anesthesiologists was summarized using Cohen’s κ (κ = 0.858). [11] Because the authors also provided the full 4 × 4 contingency table of ASA categories, we did not rely on κ-based transformations but instead recalculated the observed concordance proportion directly from the reported cell counts, treating it as a binomial accuracy measure for inclusion in the meta-analysis. The overall ASA-PS accuracy was 0.90 (2572/2851; 95% CI 0.89–0.91).

In Çelik et al., three chatbot models (ChatGPT, Copilot, Gemini) were evaluated on the same 72 patients. [30] To avoid double-counting, we derived the single study-level accuracy by averaging the model-specific AI–clinician agreement proportions and converting this mean back into an effective number of concordant classifications. We calculated single-arm concordance (k_sa_) between the three models and anesthesiologist and then calculated their single-arm accuracy (p_sa_ = k_sa_/n_tot_) separately. Then, we calculated the mean accuracy (p_m_) as p_m_ = (k_sa1_ + k_sa2_ + k_sa3_)/n_tot_, where n_tot_ = 72. Thus, if one of the three chatbots is chosen at random, the average probability that it will agree with the anesthesiologist is p_m_.

**Figure 2:**
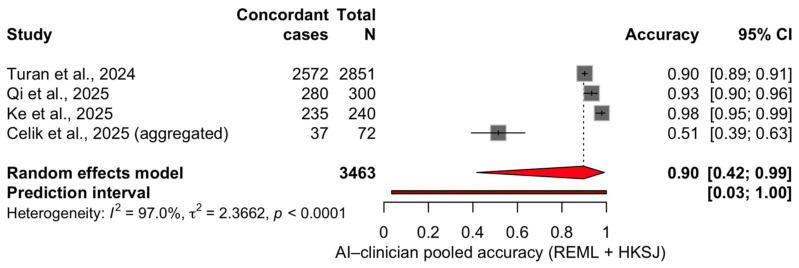
The random-effects model meta-analysis performed with REML and Hartung–Knapp–Sidik–Jonkman. The pooled accuracy of AI–clinician concordance was 0.90 (95% CI 0.42 to 0.99) with a wide 95% prediction interval (0.03 to 1.00) and high heterogeneity (I^2^ = 97%, *p* < 0.0001), indicating substantial variability in expected performance across settings. Only chatbot-based studies (N = 3607) were entered into the primary meta-analysis; non-chatbot LLM studies (Alba et al., Chung et al.) are presented as descriptive complementary evidence [24,34]. REML: restricted maximum likelihood; HKSJ: Hartung–Knapp–Sidik–Jonkman [11,25,28,30].

Using a random-effects model with REML estimation of between-study variance and Hartung–Knapp–Sidik–Jonkman confidence intervals, the pooled accuracy was 0.90 [95% CI: 0.42–0.99; 95% prediction interval 0.03–1.00] with high heterogeneity (I^2^ = 97%, *p* < 0.0001), confirming substantial variability in expected performance across studies [Figure 2].

#### 3.5.1. Subgroup Analysis: ASA Status Prediction and Perioperative Risk Stratification

Both subgroups were analyzed using REML and Hartung–Knapp–Sidik–Jonkman. The ASA status prediction subgroup (2 studies, n = 3151) reached a pooled accuracy of 0.91 (95% CI: 0.46 to 0.99) with moderate–high heterogeneity (I^2^ = 67%, τ^2^ = 0.06, *p* = 0.08). A test for linear trends across ASA complexity levels was performed and yielded a slope of −8.2 percentage points per ASA category (*p* < 0.001), indicating a statistically significant decline in accuracy with increasing patient complexity. The decline in performance from ASA I-II to ASA III is estimated to be 4.4% (4.9% relative decline, *p* = 0.31, not significant), while the decline from ASA I-II to ASA IV-V (−16.5%, 18.7% relative decline, *p* = 0.009) and from ASA III to ASA IV-V (−12.2%, 14.5% relative decline, *p* = 0.047) is statistically significant.

**Figure 3:**
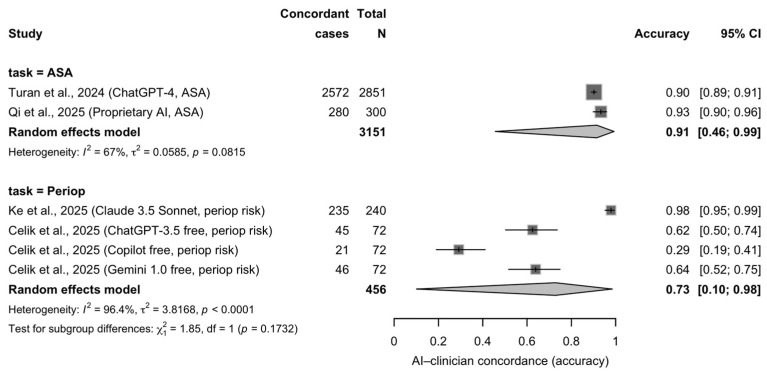
Random-effects model subgroup explorative analysis performed with REML and Hartung–Knapp–Sidik–Jonkman. ASA status prediction subgroup (n = 3151) reached a pooled accuracy of 0.91 (95% CI: 0.46 to 0.99) with moderate–high heterogeneity (I^2^ = 67%, τ^2^ = 0.06, *p* = 0.08), while perioperative risk stratification subgroup (n = 456) reached a pooled accuracy of 0.73 (95% CI: 0.10 to 0.98) with very high heterogeneity (I^2^ = 96.4%, τ^2^ = 3.8, *p* < 0.0001). Only chatbot-based studies (N = 3607) were entered into the primary meta-analysis; non-chatbot LLM studies (Alba et al., Chung et al.) are presented as descriptive complementary evidence. [23,24] REML: restricted maximum likelihood; HKSJ: Hartung–Knapp–Sidik–Jonkman [11,25,28,30].

Two studies were included in the perioperative risk stratification subgroup analysis, with a total of four different chatbots evaluated (n = 456). [11,25] The pooled accuracy was 0.73 (95% CI: 0.10 to 0.98) with very high heterogeneity (I^2^ = 96.4%, τ^2^ = 3.8, *p* < 0.0001) [Figure 3]. In this exploratory subgroup analysis by task and chatbot model, the three model-specific arms from Çelik et al. (ChatGPT, Copilot, Gemini) were displayed as separate estimates to illustrate between-model variability in perioperative risk stratification. [30] Because these arms are based on the same 72-patient cohort, their estimates are statistically correlated and are therefore interpreted descriptively only, without contributing to any additional pooled effect.

#### 3.5.2. Sensitivity Test

**Figure 4:**
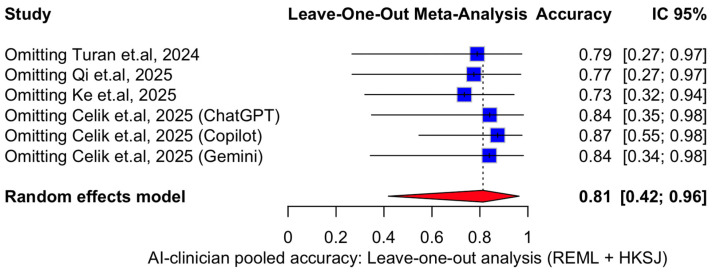
Leave-One-Out Analysis: the pooled accuracy of AI–clinician concordance remained between 0.73 and 0.87 in all iterations, with wide overlap of the confidence intervals, confirming the robustness of the random-effects model (REML + HKSJ). Omitting Celik et al. (Copilot) yielded the highest pooled accuracy estimate (0.87), indicating that this study exerts a small downward influence on the overall pooled accuracy. REML: restricted maximum likelihood; HKSJ: Hartung–Knapp–Sidik–Jonkman [11,25,28,30].

The exclusion of each study individually with recalculation of the pooled estimate through a Leave-One-Out analysis showed minimal variation in pooled accuracy and confidence interval, indicating that findings are not substantially influenced by any single study, as shown in Figure 4. By omitting Celik et al. (Copilot), the model shows the best performance (accuracy 0.87, 95% CI: 0.55 to 0.98), while by omitting Ke et al. the model shows the worst performance (accuracy 0.73, 95% CI: 0.32 to 0.94) [Figure 4]. [28,30].

### 3.6. Non-Poolable Outcomes

Because of different study design or endpoints, some outcomes were narratively analyzed.

#### 3.6.1. Risk Discrimination for Complication Prediction (AUROC, F1/F1 and MAE)

Alba et al., in their large retrospective study of 84,875 patients, developed and validated a foundation model for perioperative risk prediction [24]. The model achieved an AUROC of 0.865 (95% CI: 0.799–0.936) for predicting major perioperative complications. At the optimal cutoff threshold, the model demonstrated a sensitivity of 78.4% and specificity of 82.1%, with a positive predictive value of 31.2% and a negative predictive value of 97.3%.

Chung et al. evaluated the general-purpose LLM GPT-4 Turbo (OpenAI) accessed via an API on eight perioperative prediction tasks using task-specific datasets constructed from preoperative electronic health record notes [23]. Outcomes included ASA-PS (categorical), hospital admission, ICU admission, unplanned admission, in-hospital mortality, and three duration endpoints (PACU phase-1 time, hospital length of stay, ICU length of stay). For classification tasks, all prompt strategies outperformed a random baseline, with the best prompt achieving F1 ≈ 0.50 for ASA-PS prediction, 0.64 for hospital admission, 0.81 for ICU admission, 0.61 for unplanned admission, and 0.86 for hospital mortality. Few-shot and chain-of-thought prompting generally improved performance for ASA-PS and hospital admission, while ICU admission performance was consistently high across prompting strategies, and gains from more complex prompting were limited for unplanned admission and mortality. In contrast, numerical predictions of PACU duration, hospital length of stay, and ICU length of stay remained close to the dummy regressor baseline, with MAE around 49 min for PACU phase 1 duration, 4.5 days for hospital stay, and 1.1 days for ICU stay, and inspection of prediction distributions showed quantization and ceiling effects. Because this study used an LLM deployed purely via back-end API, reported F1 scores rather than accuracy proportions, and focused on multiple heterogeneous endpoints, we did not include it in the pooled meta-analysis; instead, we consider it complementary evidence that general-domain LLMs can achieve moderate discriminative performance for perioperative classification tasks but are not yet reliable for numerical duration prediction.

#### 3.6.2. Complication Prediction Improvement (Odds Ratio)

Ferré et al. conducted a randomized controlled trial of 389 patients comparing AI-assisted preoperative assessment versus standard assessment [26]. The trial reported that AI assistance improved the identification of patients who subsequently developed perioperative complications with an odds ratio of 21.8. The AI-assisted group identified 87.5% of patients who developed complications, compared to 42.3% in the standard assessment group, representing an absolute improvement of 45.2%.

#### 3.6.3. Decision Quality Assessment Among Different Study Design

Ruan et al. conducted a cross-sectional validation study of 30 perioperative cases evaluating the quality of clinical reasoning demonstrated by multiple LLMs [31]. Expert panels rated the overall quality of AI-generated reasoning on a 0–60 scale: compared to the human benchmark, models reached a mean performance of 74.5%, ranging from 85.5% to 59.7% (ChatGPT4: 51.3/60, 85.5%; Claude 2: 48.7/60, 81.2%; Gemini Pro: 42.1/60, 70.2%; Free models: 35.8/60, 59.7%; human expert baseline: 54.2/60, 90.3%). This demonstrates that ChatGPT-4 approaches but does not fully match human expert quality, with performance that declines substantially for less sophisticated models.

Similarly, Cheng et al. conducted a simulation-based study evaluating AI-generated recommendations for perioperative assessment and day surgery eligibility using 150 hypothetical patient scenarios [29]. Agreement between AI recommendations and expert consensus was 47.9% (95% CI: 40.1–55.8%, κ = 0.31), indicating only fair agreement. The AI system demonstrated a conservative bias, recommending inpatient surgery in 38.7% of cases compared to 24.0% for expert panels. At the optimal decision threshold, the AI achieved a sensitivity of 68.3% and specificity of 82.1% for appropriate case mix decisions.

Finally, in the study by Malek et al., ChatGPT-4.0 generated preoperative anesthetic plans for ten complex vignettes which were compared with plans from eight senior anesthesiologists and qualitatively appraised by an adjudication committee [32]. Agreement on perioperative risk assessment was limited: concordance with consultants was only fair for cardiac risk (κ = 0.41) and poor for pulmonary risk (κ = 0.13), and GPT’s risk estimates were judged appropriate in just 3/10 cases. Across cases, GPT showed a strong, repetitive preference for general anesthesia, rarely recommending neuraxial or regional techniques or multimodal analgesia, and often diverged from guideline-consistent practice. Overall, experts concluded that GPT-generated plans were generally coherent but frequently failed to meet minimum standards of good clinical care and did not appear to reflect genuine clinical reasoning. ChatGPT-4.0 was indeed not considered suitable for preoperative planning in its current form.

### 3.7. GRADE

Overall, the GRADE assessment indicates low to very low certainty of evidence regarding chatbot-assisted perioperative risk stratification. Current data suggest that LLM-based chatbots may be considered only clinician-supervised decision support tools for ASA I–III patients in routine settings, while autonomous use in complex cases or general perioperative risk stratification remains inappropriate [Table 7].

**Figure 5:**
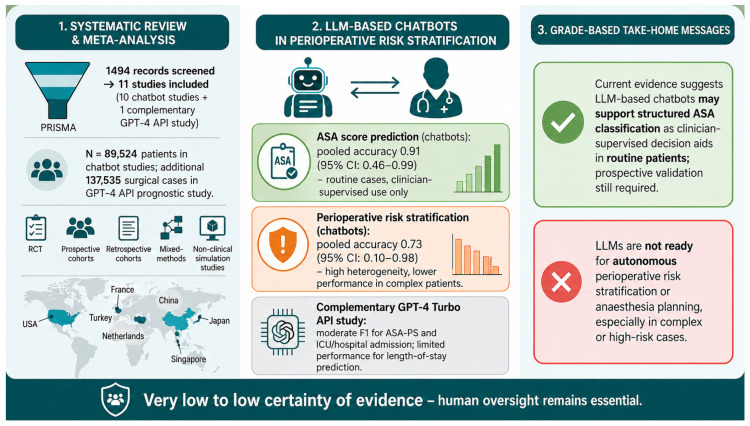
Synthesis of results.

Based on this low-certainty evidence, potential implications for clinical use can be summarized as follows [Figure 5]:

1. Chatbots act as a decision support tool in risk stratification (not suitable for autonomous decision-making).
2. They are used for classification tasks (they struggle in unstructured tasks).
3. Use is restricted to structured classification tasks (ASA I–III) where evidence shows adequate performance, in particular with proprietary models.
4. Clinical oversight and judgment remain central to the decision process.
5. Results should always be reviewed by an attending anesthesiologist.
6. Use is not recommended for complex cases (ASA IV–V) or autonomous planning.
7. The limitations of the technology are clearly understood by users.

These recommendations reflect the risk stratification of the use of chatbots if applied in clinical practice:

- Routine cases (ASA I–III): small harm risk if used as decision support.
- Complex cases (ASA IV–V): serious harm risk if used autonomously.
- Autonomous use: unacceptable harm risk (>33% unsafe recommendations).

The very low quality of evidence reflects the current state of research on chatbots in perioperative risk stratification, characterized by observational studies with methodological limitations, heterogeneous methods and outcomes, and small sample sizes.

## 4. Discussion

This paper represents the first systematic review investigating the real-world implementation of chatbots for perioperative risk stratification and anesthesia management. We evaluated the performance of widely used chatbots, focusing on their application in preoperative surgical patient assessment, and assessed both the benefits and harms of chatbot integration in clinical practice using quantitative and qualitative evaluations.

Although these state-of-the-art AI systems cannot yet be considered validated tools for routine clinical use, as noted by Daccache et al., our findings highlight a potential application during the preoperative period, consistent with the current literature [23,34].

Our analysis indicates that chatbot-assisted ASA classification can achieve accuracy comparable to clinicians for routine ASA I–III patients, potentially offering efficiency gains. However, performance declines markedly in complex cases, with a substantial reduction in accuracy for ASA IV–V patients and occasional unsafe recommendations in anesthesia planning. The meta-analysis demonstrates significant inter-study variability, largely explained by the type of LLM, patient complexity, different clinical settinsg and endpoints and an accuracy that declines progressively from ASA I–II to ASA IV–V patients, with ChatGPT-4 maintaining high performance in lower and intermediate classes, while performance drops primarily in the highest-risk patients. Free models exhibit earlier and steeper losses of concordance. The gap between proprietary and free models widens with increasing complexity. Sensitivity analysis, in particular Leave-One-Out Analysis, shows no substantial difference, confirming that the evidence is consistent with a moderately high effect, but with considerable uncertainty. This indicates the robustness of the findings but does not rule out residual heterogeneity since the CIs remain wide and the overall precision is still limited.

Historically, AI’s clinical impact in anesthesia has been limited to data display and analysis, but adoption is increasing as clinicians better understand its potential and limitations [35]. Chatbots offer several promising functions, including knowledge enhancement, clinical decision support, patient education and engagement, and time optimization through administrative automation, such as drafting agendas, summarizing medical histories, or generating medical documents [36].

Despite these advances, the literature addressing chatbot applications in perioperative care remains scarce. The most extensively validated use is in patient education, where chatbots deliver personalized information, facilitate bidirectional communication, and promote active patient participation in care [37].

Lin et al. recently conducted a systematic review of perioperative chatbot applications, mainly focusing on patient education, communication quality and patient-centered outcomes such as satisfaction and decision regret [14]. Across eight studies involving 1073 adults, they found that approximately three-quarters of patients were satisfied with chatbot-supported perioperative care and around four-fifths reported improved procedural knowledge. The included interventions were predominantly rule-based, targeted mainly orthopedic and pre-anesthesia settings, and did not report chatbot-related harms, although study quality was generally only fair and outcomes were heterogeneous. Their synthesis showed that chatbots can improve information delivery and patient engagement, but did not quantitatively evaluate perioperative risk stratification or anesthesia planning performance. Our review specifically addresses this gap by focusing on LLM-based chatbots used for ASA classification, complication prediction and anesthesia decision-making [14].

Our pooled accuracy estimates exceed those reported for traditional ML approaches. Wongtangman et al. reported 57.2% agreement between ML-predicted and clinician-assigned ASA scores in 337,481 patients [38], while Lew et al. achieved 69.6% accuracy using deep learning on 12,064 patients [39]. These findings suggest that large language models pre-trained on diverse biomedical corpora may provide substantial advantages over conventional feature-based ML approaches.

### 4.1. Recommendations for Clinical Implementation and Safe Clinical Integration Guidelines

A critical limitation is performance deterioration in high-risk patients. For ASA IV–V patients, accuracy declines sharply, and approximately one-third of chatbot-generated anesthesia plans were deemed unsafe by expert review. This highlights a divergence between pattern recognition, in which models correctly assign ASA classes, and clinical reasoning, which is required for safe anesthesia planning. While chatbots demonstrate utility in categorical or binary prediction tasks, such as interpreting EHR data into structured judgments, they remain unreliable for precise numerical prognostication like hospital length of stay, and they lack bidirectional interactivity necessary for comprehensive preoperative assessment [28].

From a technical perspective, prompt engineering is essential to optimize AI performance, particularly in complex tasks. Instructions should be clear, precise, and contextually complete, using simple and direct language while avoiding unnecessary complexity. Iterative refinement of prompts may be required to achieve optimal results [40]. Explainability is equally critical. Systems such as GPT-4 Turbo provide not only risk predictions but also high-quality natural language explanations, enhancing clinician trust and understanding [28]. Accountability and governance must be addressed. Errors in AI recommendations pose complex liability challenges, necessitating clear policies and legal frameworks to define responsibility. Structured feedback mechanisms allow clinicians to learn from AI-related errors, whether arising from adherence to or deviation from AI recommendations [41].

In addition, chatbot efficacy depends on high-quality pre-training datasets, including medical-specific language, regular updates, and retraining. Real-time internet access may improve reliability but raises copyright concerns. Current models rely primarily on open-access sources such as research papers and podcasts, whereas higher accuracy requires EHR or institutional data validated by anesthesiologists.

Although GPT-4, Bard, and Bing Chat excel in anesthesia-related communication, the accuracy of medical content remains suboptimal [42]. Future RCTs are essential to optimize chatbot platforms for perioperative applications [15]. Tools like MyRISK, which collect self-reported patient data before pre-anesthetic consultation, demonstrate potential for teleconsultation triage, though broader validation is needed [26].

Overall, anesthesia departments should restrict chatbot use to routine ASA I–III classifications and explicitly prohibit autonomous use in complex cases. Structured clinician training on technology limitations is recommended, alongside implementation of audit and quality assurance mechanisms. Governance frameworks should clarify AI authority, responsibilities, and liability, and clinician review of all recommendations should be mandatory before implementation. Chatbots should remain optional support tools within well-defined boundaries.

### 4.2. Limitations

This review has several limitations. We focused on LLM-based chatbots rather than LLMs accessed only via APIs, as usability is central to clinical deployment; however, this does not exclude that non-chatbot LLMs might perform similarly on the same tasks [16]. For this reason, studies evaluating non-chatbot models were not included in the primary meta-analysis. For example, Chung et al. (2023) evaluated a model powered by OpenAI API rather than a chatbot interface [43]. In addition, the evaluated outcome (F1 score) could not be pooled together with different outcome measures within the same meta-analysis. We restricted inclusion to LLM-based systems deployed as bidirectional chatbots, because usability and interaction modality are central to their potential clinical implementation. Studies evaluating LLMs exclusively via application programming interfaces (APIs) without a conversational interface were therefore excluded by design.

Our quantitative synthesis has methodological limitations that warrant caution. First, in the study by Çelik et al., three chatbot models (ChatGPT, Copilot, Gemini) were evaluated on the same cohort of 72 orthopedic patients. [30]. To avoid double-counting the same cases in the main meta-analysis, we derived a single aggregated accuracy estimate by averaging the model-specific AI–clinician agreement proportions and converting this mean back into an ‘effective’ number of concordant classifications. This pragmatic strategy reduces, but does not eliminate, the dependence between model-level estimates and should be interpreted as an approximation of the overall performance of the three chatbots in that specific setting. In addition, in exploratory subgroup analyses, we display the three model-specific accuracies from Çelik et al. as separate points to illustrate between-model variability [30]. These estimates are inherently correlated because they are based on the same patient sample; therefore, subgroup comparisons involving these three arms should be interpreted descriptively rather than as independent confirmatory evidence.

Most included studies were observational or based on simulated cases, with heterogeneous endpoints and performance metrics. This limited the feasibility of a comprehensive meta-analysis and precluded a formal assessment of publication bias, despite the inclusion of gray literature. Although a pattern suggestive of publication bias was observed, Egger’s test could not be performed due to the small number of studies included.

The evidence base is further constrained by geographic concentration in a single country, potential language bias, and underrepresentation of elderly, emergency, and rural populations. Additional variability arises from differences in chatbot training, ranging from domain-specific systems to general-purpose models such as ChatGPT, alongside a predominance of studies reporting favorable results.

Several methodological features deserve attention. In some studies, case selection excluded more complex patients or used simplified scenarios, and one investigation relied on examination questions instead of real cases, which may limit generalizability. Outcome assessment sometimes relied on composite complications collected by telephone at six months or on rare events such as pulmonary embolism and death, making estimates sensitive to recall and class imbalance. Approaches to confounding also varied: seven studies offered limited adjustment or comparison groups, whereas the non-inferiority RCT by Qi et al. and the multicenter study by Turan et.al incorporated more structured control of confounders [11,25]. Prospective registration was uncommon, with only one trial preregistered and another registered after enrolment had begun.

### 4.3. Future Directions

This review introduces a novel clinical perspective by integrating evidence across previously fragmented domains—artificial intelligence, perioperative risk stratification, and anesthesia decision-making—while applying a combined methodological framework of systematic review, meta-analysis, and GRADE-based evidence. Unlike prior reviews, which primarily focused on patient education, user experience, and communication quality, this study delivers a quantitative assessment of clinical performance, including accuracy, concordance, and risk prediction, with direct comparison to clinician judgment and subgroup analyses by model type and patient complexity. In doing so, it identifies critical gaps and inconsistencies, particularly the marked decline in performance in high-risk scenarios, and organizes the current evidence into a coherent framework for clinical interpretation. Importantly, this review translates findings into real-world implications by defining clear boundaries for safe implementation: supporting the use of chatbots as supervised decision support tools in structured, low-risk tasks while discouraging their use in complex clinical decision-making. These insights provide actionable guidance for clinicians, inform policy and governance frameworks, and highlight priorities for future research and responsible integration of AI into perioperative practice.

Future studies should include prospective designs with adequate sample sizes, standardized outcome measures, and direct comparisons with clinician judgment across diverse populations, with explicit evaluation of real-world clinical outcomes. Domain-specific models trained on biomedical data appear more promising than general-purpose LLMs and should be prioritized for further development.

Priority areas include large trials across multiple surgical specialties, systematic assessment of clinician satisfaction and workflow integration, and rigorous testing of chatbots’ ability to estimate comprehensive perioperative risk, support anesthesia decisions, and handle both traditional and machine learning risk scores in real time. Domain-specific models trained on biomedical corpora (for example, BioGPT or BioClinicalBERT) appear more promising than general-purpose LLMs, and their further development and evaluation should be a focus.

Additional gaps concern the lack of data for high-risk populations, the unquantified frequency and impact of hallucinations and false recommendations, the absence of long-term safety follow-up, the limited head-to-head comparisons with senior anesthesiologists, and the complete lack of validation in low- and middle-income countries, where such tools might be most needed. Future research should therefore emphasize prospective registration, representative case selection, blinded outcome assessment, external validation in different institutions and health systems, and transparent reporting of both benefits and harms.

## 5. Conclusions

This systematic review represents the first comprehensive evidence synthesis on chatbot-assisted perioperative risk stratification and is the first to establish a GRADE-based certainty of evidence assessment. LLM-based chatbots show promising performance in routine perioperative risk stratification but remain unreliable in complex cases, with relevant safety concerns. Given the very low certainty of evidence, these tools should, at most, be used as clinician-supervised decision support aids for routine ASA assessment and should not be relied upon for autonomous use in complex cases or for general perioperative risk stratification.

## Figures and Tables

**Table 1:** PICO framework.

P—Population	I—Intervention	C—Comparison	O—Outcome
Adult patients undergoing surgical procedures requiring anesthesia and perioperative anesthesiologic assessment	Chatbots powered by large language models (LLMs) for perioperative risk stratification and anesthesia planning	Standard clinical reference: traditional clinical judgment of anesthesiologists and physicians in preoperative assessment and anesthetic planning (in-person evaluation)	Primary: chatbot performance in risk stratification in terms of the area under the receiver operating characteristic (AUROC), accuracy, specificity, sensitivity, Cohen’s Kappa, and percent agreement. Secondary: assessment time, documentation quality, safety (hallucinations, deviations), and patient satisfaction

**Table 2:** Sample size of included studies ordered by number of observations (N).

Author	N	% of Total	Study Design	Country
Chung et al. [23]	137,535	60.57%	Retrospective cohort	USA
Alba et al. [24]	84,875	37.38%	Retrospective cohort	USA
Turan et al. [11]	2851	1.26%	Prospective	Turkey
Qi et al. [25]	600	0.26%	Randomized Controlled Trial, Non-Inferiority	China
Ferré et al. [26]	401	0.18%	Prospective	France
Fujimoto et al. [27]	295	0.13%	Comparative, non-clinical	Japan
Ke et al. [28]	240	0.11%	Prospective	Singapore
Cheng et al. [29]	150	0.07%	Case–control	China
Çelik et al. [30]	72	0.03%	Prospective	Turkey
Ruan et al. [31]	30	0.01%	Prospective	China
Malek et al. [32]	10	>0.01%	Mixed-method exploratory	Netherlands
TOTAL	227,059	100.00%	—	—

**Table 3:** The proportion of population characteristics and surgery type in the included studies (where reported). Population comes from tertiary/university hospitals and some community and day-surgery units.

Patient Characteristics	Summary
Sex	46–55% (balanced male/female distribution)
Mean age	45–58 years
ASA I–II	34.5%
ASA III	46.2%
ASA IV–V	19.2%
Surgery type (where reported)	Summary
General surgery	38%
Orthopedic surgery	22%
Gynecology	15%
Urology	12%
Other/mixed	13%

**Table 4:** Geographical distribution of included population.

Country	N	% of Total	Number of Studies
North America (USA)	222,410	97.9%	2
Asia (Middle East, Turkey)	2923	1.3%	2
Asia (China)	780	0.3%	3
Europe (France)	401	0.2%	1
Asia (Japan)	295	0.1%	1
Asia (Singapore)	240	0.1%	1
Europe (Netherlands)	10	>0.01%	1
TOTAL	227,059	100%	11

**Table 5:** Risk of bias assessment of included studies. Most frequent limitations were selection bias, confounding in non-randomized comparisons, and outcome/reporting limitations. PROBAST highlighted concerns mainly in analysis and outcome domain.

Tool	Study Design	Domain-Level Risk of Bias (Tool-Specific)	Overall RoB	Applicability Concerns for Perioperative Clinical Use
PROBAST-AI	Alba 2025 (retrospective; N = 84,875) [24]	Participants/data: U; Predictors: U; Outcome: H (e.g., outcome missingness noted for delirium); Analysis: H (rare events/imbalance + limited transportability details).	High	Low–Moderate (clinical population/outcomes aligned, but internal validity issues).
PROBAST-AI	Ferré 2023 (prospective; MyRISK digital stratification; N = 401) [26]	Participants/data: U; Predictors: U; Outcome: U; Analysis: H (small sample, limited validation reporting).	High	High (restricted day-surgery orthopedic setting and exclusions reduce generalizability).
RoB-2	Qi 2025 (RCT non-inferiority; AI-assisted preop evaluation; N = 600) [25]	Randomization: M; Deviations: U; Missing outcome data: U; Outcome measurement: M; Selective reporting: M (registration/reporting concerns).	Moderate	Low (direct clinical workflow study in surgical patients).
ROBINS-I	Chung 2024 (retrospective; GPT-4 Turbo API; task-specific datasets, n ≈ 1000 per outcome) [23]	Participants/data: M; Predictors: U; Outcome: U; Analysis: M-H	Moderate–High	Moderate–High (real surgical population and relevant outcomes, but implementation as GPT-4 Turbo via back-end API only, without a clinician-facing chatbot or direct integration into perioperative workflows).
ROBINS-I	Turan 2024 (prospective multicenter; ASA vs. clinicians; N = 2851) [11]	Confounding: L; Selection: L; Outcomes: M; Reporting: L	Low–Moderate	Low
ROBINS-I	Çelik 2025 (prospective; anesthesia-method concordance; N = 72) [30]	Confounding: M; Selection: M; Outcomes: M; Reporting: H	Moderate–High	High (small sample size, restricted case mix).
ROBINS-I	Ruan 2025 (prospective; complex-case LLM scoring; N = 30) [31]	Confounding: M; Selection: H; Outcomes: M; Reporting: H	Moderate–High	High (selected complex vignettes/scenarios; very small sample size).
ROBINS-I	Cheng 2024 (case–control; simulated presentations; N = 150) [29]	Confounding: M; Selection: H; Outcomes: M; Reporting: H	Serious	High (simulated patients).
ROBINS-I	Fujimoto 2024 (non-clinical; board exam questions; N = 295) [27]	Confounding: H; Selection: H; Outcomes: H; Reporting: H	Serious	High (proxy outcome, not perioperative patient care).
ROBINS-I	Ke 2025 (prospective; PEACH, N reported as 240/272) [28]	Confounding: H; Selection: H; Outcomes: H; Reporting: H	Serious	High (population and endpoint framework limit direct clinical applicability).
ROBINS-I	Abdel Malek 2024 (mixed method; vignettes; N = 10) [32]	Confounding: H; Selection: H; Outcomes: H; Reporting: H	Serious	High (vignettes and very small sample size).

**Table 6:** Systematic review results table following Cochrane guidelines showing the description, characteristics and results of the included articles listed by study design and year. Abbreviations: LLM: large language model; AI: artificial intelligence; ASA: American Society of Anesthesiologists Physical Status; RoB: risk of bias. † Non-chatbot LLM studies: included as complementary evidence only; excluded from pooled meta-analysis.

Author	Year	Country	Design	N	Setting	AI Model	Primary Outcome	Comparator	RoB
Ferré et al. [26]	2023	Spain	Prospective cohort	401	University hospital	Medical Assistant Experience (MAX)	Complication identification improvement	Blinded expert review	Low RoB
Qi et al. [25]	2025	China	Prospective cohort (randomized non-inferiority trial)	600	Tertiary teaching hospital	Proprietary model	ASA classification accuracy	Single expert anesthesiologist	Medium RoB
Ke et al. [28]	2025	China	Prospective cohort	240	University teaching hospital	Claude 3.5 Sonnet	Perioperative risk stratification	Guidelines and expert panel	Medium RoB
Malek et al. [32]	2025	Netherlands	Mixed-method exploratory	10	Academic medical center	ChatGPT-4	ASA classification accuracy	Expert consensus	High RoB
Çelik et al. [30]	2025	Turkey	Prospective cohort	72	Community hospital	ChatGPT, Copilot e Gemini	Perioperative risk stratification—type of anesthesia	Staff anesthesiologist	Medium RoB
Turan et al. [11]	2024	Turkey	Prospective cohort	2851	University hospital	ChatGPT-4	ASA classification accuracy	Expert consensus panel (3 experts)	Medium RoB
† Alba et al. [24]	2025	USA	Retrospective cohort	84,875	Multi-center (5 hospitals)	Foundation model (BioGPT, ClinicalBERT, BioClinicalBERT)	AUROC for complication prediction	Actual perioperative outcomes	Low RoB
Ruan et al. [31]	2025	China	Cross-sectional validation	30	Academic center	Multiple LLMs	Decision quality score	Expert panel (3 reviewers)	High RoB
Fujimoto et al. [27]	2024	Japan	Cross-sectional, non-clinical comparative study	295	Board exam setting (no real patients)	ChatGPT-4, Claude 3 Opus, Gemini 1.0	Correct answer rate on dental anesthesiology exam questions	Official exam key	High RoB
Cheng et al. [29]	2025	China	Simulation-based	150	Simulated scenarios	ChatGPT-4	Agreement with expert recommendations	Expert consensus	High RoB
† Chung et al. [23]	2024	USA	Retrospective prognostic study	137,535	Three academic hospitals and affiliated clinics	GPT-4 Turbo (OpenAI API)	F1 score for perioperative risk prediction tasks (ASA-PS, ICU/hospital admission, unplanned admission, in-hospital mortality)	Ground-truth outcomes from EHR	Moderate–High RoB

**Table 7:** GRADE group evidence table. ^a^ The pooled estimate comes from one randomized non-inferiority trial and three prospective non-randomized cohort studies. [11,28,29,30] We entered the meta-analytic result under ‘non-randomized studies’ and rated certainty starting from low, even though Qi et al. has an RCT design. [25] Bias rate derives from observational studies with methodological limitations, heterogeneous methods and outcomes, and small sample sizes. High risk of confounding, selection bias, publication bias and reporting issues. ^b^ A very wide CI in the random-effect model. ^c^ Overall I^2^ > 75%. ^d^ Relative risk not estimable. Traditional clinical judgment is the benchmark. CI: confidence interval.

Certainty Assessment							№ of Patients		Effect		Certainty	Importance
№ of Studies	Study Design	Risk of Bias	Inconsistency	Indirectness	Imprecision	Other Considerations	Chatbots Powered by Large Language Models (LLMs)	Traditional Clinical Judgment of Anesthesiologists	Relative (95% CI)	Absolute (95% CI)		
Global AI–clinician concordance (assessed with accuracy)												
4 ^a^	non-randomized studies	very serious ^a^	very serious ^b,c^	not serious	serious ^b^	^a^	3199/3607 (88.7%)	3607/3607 (100.0%)	not estimable ^d^	0.81 (0.96 to 0.42) ^d^	Very Low	CRITICAL
ASA status prediction (assessed with accuracy)												
2 ^a^	non-randomized studies	moderate ^a^	not serious	not serious	serious ^b^	^a^	2852/3151 (90.5%)	3151/3151 (100.0%)	not estimable ^d^	0.91 (0.46 to 0.99) ^d^	Low	CRITICAL
Perioperative risk prediction (assessed with accuracy)												
2 ^a^	non-randomized studies	very serious ^a^	very serious ^b,c^	not serious	very serious ^b^	^a^	347/456 (76.1%)	456/456 (100.0%)	not estimable ^d^	0.73 (0.10 to 0.98) ^d^	Very Low	CRITICAL

## Data Availability

Data are available upon request.

## Supplementary Materials

The following supporting information can be downloaded at https://www.mdpi.com/article/10.3390/jcm15124670/s1, File S1: Search strategies; Table S1: Extended Preferred Reporting Items for Systematic Reviews and Meta-analyses (PRISMA) 2020 statement with PRISMA Abstract Checklist; Table S2: PRISMA extension for Reporting Literature Searches (PRISMA-S).

## Abbreviations

The following abbreviations are used in this manuscript:

AI	Artificial Intelligence
AKI	Acute Kidney Injury
ASA	American Society of Anesthesiologists Physical Status
ASA-PS	American Society of Anesthesiologists Physical Status Score
AUROC	Area Under the Receiver Operating Characteristic Curve
AUPRC	Area Under the Precision–Recall Curve
CDS	Clinical Decision Support
CI	Confidence Interval
DL	Deep Learning
DVT	Deep Vein Thrombosis
EHR	Electronic Health Record
EtD	Evidence-to-Decision (framework)
GAI	Generative Artificial Intelligence
GAIDeT	Generative AI Delegation Taxonomy
GRADE	Grading of Recommendations, Assessment, Development and Evaluation
HKSJ	Hartung–Knapp–Sidik–Jonkman (method)
I^2^	Inconsistency statistic (heterogeneity measure)
LLM	Large Language Model
LLMs	Large Language Models
MAE	Mean Absolute Error
MeSH	Medical Subject Headings
MINORS	Methodological Index for Non-Randomized Studies
ML	Machine Learning
NLP	Natural Language Processing
NSQIP	National Surgical Quality Improvement Program
PEACH	PErioperative AI CHatbot
PICO	Population, Intervention, Comparator, Outcome (framework)
PLM	Pre-trained Language Model
PRISMA	Preferred Reporting Items for Systematic Reviews and Meta-Analyses
PRISMA-S	PRISMA extension for Reporting Literature Searches
PROBAST-AI	Prediction Model Risk of Bias Assessment Tool—Artificial Intelligence extension
PRS	Perioperative Risk Stratification
PROSPERO	International Prospective Register of Systematic Reviews
RCT	Randomized Controlled Trial
REML	Restricted Maximum Likelihood
RoB	Risk of Bias
RoB-2	Risk of Bias tool 2 (for randomized trials)
ROBINS-I	Risk of Bias in Non-randomized Studies—of Interventions
SD	Standard Deviation
SORT	Surgical Outcome Risk Tool
τ^2^	Tau-squared (between-study variance)
UFF	Universidade Federal Fluminense
WHO ICTRP	World Health Organization International Clinical Trials Registry Platform
