# Supplementary material for: Perioperative Risk Stratification with AI-Powered Chatbots: A Systematic Review and Meta-Analysis
Source: J Clin Med. 2026 Jun 16;15(12):4670. doi: 10.3390/jcm15124670 (PMC13301336; doi:10.3390/jcm15124670)
Supplement: Supplementary file 1 [file jcm-15-04670-s001.zip › File S1.pdf]

Supplementary File S1 – Search strategies

Last search run: January 2026

Database: PubMed (MEDLINE via PubMed)

1. chatbot[all] AND ("perioperative assessment"[all] OR "preoperative evaluation"[all] OR "anesthesia evaluation"[all])

2. (("Artificial Intelligence"[Mesh] OR "artificial intelligence"[all] OR AI[all]) AND chatbot[all] AND (anesthesia[all] OR anesthesiology[all]))

3. "conversational agent"[all] AND ("perioperative care"[all] OR "preoperative screening"[all] OR "anesthesia consultation"[all])

4. ("virtual assistant"[all] OR "digital assistant"[all]) AND (anesthesia[all] OR perioperative[all])

5. "natural language processing"[all] AND anesthesia[all] AND (preoperative[all] OR perioperative[all])

6. ("machine learning"[all] OR "deep learning"[all]) AND chatbot[all] AND ("anesthesia risk assessment"[all] OR "preoperative evaluation"[all])

7. "automated conversation"[all] AND (anesthesia[all] OR "perioperative care"[all] OR "preoperative screening"[all] OR "perioperative assessment"[all])

8. "intelligent virtual agent"[all] AND (anesthesiology[all] OR "perioperative care"[all] OR "preoperative assessment"[all])

9. (("Artificial Intelligence"[Mesh] OR "artificial intelligence"[all] OR chatbot[all] OR chatgpt[all]) AND "revised cardiac risk index"[all])

10. (("Frailty"[Mesh] OR frailty[all] OR "frailty index"[all] OR "clinical frailty index"[all]) AND (artificial intelligence[all] OR chatbot[all] OR chatgpt[all]) AND (surgery[all] OR perioperative[all]))

11. (("Artificial Intelligence"[Mesh] OR "artificial intelligence"[all] OR chatbot[all] OR chatgpt[all]) AND "revised cardiac risk index"[all] AND (surgery[all] OR perioperative[all]))

12. (("Acute Kidney Injury"[Mesh] OR "acute kidney injury"[all] OR "AKI risk index"[all] OR "AKI prediction"[all]) AND (artificial intelligence[all] OR chatbot[all] OR chatgpt[all]) AND (surgery[all] OR perioperative[all]))

13. (chatbot[all] OR chatgpt[all]) AND (preoperative[all] OR anesthesiology[all]) AND evaluation[all]

14. (chatgpt[all] OR chatbot[all]) AND ("ASA score"[all] OR "ASA status"[all]) AND "preoperative risk assessment"[all]

15. (chatgpt[all] OR chatbot[all]) AND ("ASA score"[all] OR "ASA status"[all]) AND "preoperative risk prediction"[all]

16. (chatgpt[all] OR chatbot[all]) AND ("ASA score"[all] OR "ASA status"[all]) AND prediction[all]

17. (("Artificial Intelligence"[Mesh] OR "artificial intelligence"[all] OR "large language model"[all] OR "large language models"[all] OR chatbot[all] OR chatgpt[all]) AND (perioperative[all] OR surgery[all]) AND ("clinical frailty index"[all] OR "frailty index"[all]))

18. (("Artificial Intelligence"[Mesh] OR "artificial intelligence"[all] OR "large language model"[all] OR "large language models"[all] OR chatbot[all] OR chatgpt[all]) AND (perioperative[all] OR surgery[all]) AND NSQIP[all])

19. (("Acute Kidney Injury"[Mesh] OR "acute kidney injury"[all] OR "AKI risk index"[all]) AND ("Artificial Intelligence"[Mesh] OR "artificial intelligence"[all] OR "large language model"[all] OR "large language models"[all] OR chatbot[all] OR chatgpt[all]) AND (perioperative[all] OR surgery[all]))

20. (("Artificial Intelligence"[Mesh] OR "artificial intelligence"[all] OR "large language model"[all] OR "large language models"[all] OR chatbot[all] OR chatgpt[all]) AND (perioperative[all] OR surgery[all]) AND "Mini-Cog"[all])

21. (("Artificial Intelligence"[Mesh] OR "artificial intelligence"[all] OR "large language model"[all] OR "large language models"[all] OR chatbot[all] OR chatgpt[all]) AND (perioperative[all] OR surgery[all]) AND SORT[all])

22. (("Artificial Intelligence"[Mesh] OR "artificial intelligence"[all] OR "large language model"[all] OR "large language models"[all] OR chatbot[all] OR chatgpt[all]) AND (perioperative[all] OR surgery[all]) AND "P-POSSUM"[all])

23. (("Artificial Intelligence"[Mesh] OR "artificial intelligence"[all] OR "large language model"[all] OR "large language models"[all] OR chatbot[all] OR chatgpt[all]) AND (perioperative[all] OR surgery[all]) AND "EuroSCORE II"[all])

Database: Embase (Ovid)

1. chatbot.ti,ab. AND (perioperative assessment.ti,ab. OR preoperative evaluation.ti,ab. OR anesthesia evaluation.ti,ab.)

2. (artificial intelligence.ti,ab. OR AI.ti,ab.) AND chatbot.ti,ab. AND (anesthesia.ti,ab. OR anesthesiology.ti,ab.)

3. "conversational agent*".ti,ab. AND (perioperative care.ti,ab. OR preoperative screening.ti,ab. OR anesthesia consultation.ti,ab.)

4. ("virtual assistant*".ti,ab. OR "digital assistant*".ti,ab.) AND (anesthesia.ti,ab. OR perioperative.ti,ab.)

5. "natural language processing".ti,ab. AND anesthesia.ti,ab. AND (preoperative.ti,ab. OR perioperative.ti,ab.)

6. ("machine learning".ti,ab. OR "deep learning".ti,ab.) AND chatbot.ti,ab. AND ("anesthesia risk assessment".ti,ab. OR "preoperative evaluation".ti,ab.)

7. "automated conversation".ti,ab. AND (anesthesia.ti,ab. OR "perioperative care".ti,ab. OR "preoperative screening".ti,ab. OR "perioperative assessment".ti,ab.)

8. "intelligent virtual agent*".ti,ab. AND (anesthesiology.ti,ab. OR "perioperative care".ti,ab. OR "preoperative assessment".ti,ab.)

9. (artificial intelligence.ti,ab. OR chatbot.ti,ab. OR chatgpt.ti,ab.) AND "revised cardiac risk index".ti,ab.

10. (artificial intelligence.ti,ab. OR chatbot.ti,ab. OR chatgpt.ti,ab.) AND "frailty index".ti,ab. AND (surgery.ti,ab. OR perioperative.ti,ab.)

11. (artificial intelligence.ti,ab. OR chatbot.ti,ab. OR chatgpt.ti,ab.) AND "revised cardiac risk index".ti,ab. AND (surgery.ti,ab. OR perioperative.ti,ab.)

12. (artificial intelligence.ti,ab. OR chatbot.ti,ab. OR chatgpt.ti,ab.) AND ("AKI prediction".ti,ab. OR "acute kidney injury".ti,ab.) AND (surgery.ti,ab. OR perioperative.ti,ab.)

13. (chatbot.ti,ab. OR chatgpt.ti,ab.) AND (preoperative.ti,ab. OR anesthesiology.ti,ab.) AND evaluation.ti,ab.

14. (chatgpt.ti,ab. OR chatbot.ti,ab.) AND ("ASA score".ti,ab. OR "ASA status".ti,ab.) AND "preoperative risk assessment".ti,ab.

15. (chatgpt.ti,ab. OR chatbot.ti,ab.) AND ("ASA score".ti,ab. OR "ASA status".ti,ab.) AND "preoperative risk prediction".ti,ab.

16. (chatgpt.ti,ab. OR chatbot.ti,ab.) AND ("ASA score".ti,ab. OR "ASA status".ti,ab.) AND prediction.ti,ab.

17. ("artificial intelligence".ti,ab. OR "large language model*".ti,ab. OR chatbot.ti,ab. OR chatgpt.ti,ab.) AND (perioperative.ti,ab. OR surgery.ti,ab.) AND ("clinical frailty index".ti,ab. OR "frailty index".ti,ab.)

18. ("artificial intelligence".ti,ab. OR "large language model*".ti,ab. OR chatbot.ti,ab. OR chatgpt.ti,ab.) AND (perioperative.ti,ab. OR surgery.ti,ab.) AND NSQIP.ti,ab.

19. ("artificial intelligence".ti,ab. OR "large language model*".ti,ab. OR chatbot.ti,ab. OR chatgpt.ti,ab.) AND (perioperative.ti,ab. OR surgery.ti,ab.) AND "AKI risk index".ti,ab.

20. ("artificial intelligence".ti,ab. OR "large language model*".ti,ab. OR chatbot.ti,ab. OR chatgpt.ti,ab.) AND (perioperative.ti,ab. OR surgery.ti,ab.) AND "Mini-Cog".ti,ab.

21. ("artificial intelligence".ti,ab. OR "large language model*".ti,ab. OR chatbot.ti,ab. OR chatgpt.ti,ab.) AND (perioperative.ti,ab. OR surgery.ti,ab.) AND SORT.ti,ab.

22. ("artificial intelligence".ti,ab. OR "large language model*".ti,ab. OR chatbot.ti,ab. OR chatgpt.ti,ab.) AND (perioperative.ti,ab. OR surgery.ti,ab.) AND "P-POSSUM".ti,ab.

23. ("artificial intelligence".ti,ab. OR "large language model*".ti,ab. OR chatbot.ti,ab. OR chatgpt.ti,ab.) AND (perioperative.ti,ab. OR surgery.ti,ab.) AND "EuroSCORE II".ti,ab.

Database: Scopus

1. TITLE-ABS-KEY(chatbot AND ("perioperative assessment" OR "preoperative evaluation" OR "anesthesia evaluation"))

2. TITLE-ABS-KEY(("artificial intelligence" OR AI) AND chatbot AND (anesthesia OR anesthesiology))

3. TITLE-ABS-KEY("conversational agent" AND ("perioperative care" OR "preoperative screening" OR "anesthesia consultation"))

4. TITLE-ABS-KEY(("virtual assistant" OR "digital assistant") AND (anesthesia OR perioperative))

5. TITLE-ABS-KEY("natural language processing" AND anesthesia AND (preoperative OR perioperative))

6. TITLE-ABS-KEY(("machine learning" OR "deep learning") AND chatbot AND ("anesthesia risk assessment" OR "preoperative evaluation"))

7. TITLE-ABS-KEY("automated conversation" AND (anesthesia OR "perioperative care" OR "preoperative screening" OR "perioperative assessment"))

8. TITLE-ABS-KEY("intelligent virtual agent" AND (anesthesiology OR "perioperative care" OR "preoperative assessment"))

9. TITLE-ABS-KEY((artificial AND intelligence OR chatbot OR chatgpt) AND "revised cardiac risk index")

10. TITLE-ABS-KEY((artificial AND intelligence OR chatbot OR chatgpt) AND "frailty index" AND (surgery OR perioperative))

11. TITLE-ABS-KEY((artificial AND intelligence OR chatbot OR chatgpt) AND "revised cardiac risk index" AND (surgery OR perioperative))

12. TITLE-ABS-KEY((artificial AND intelligence OR chatbot OR chatgpt) AND ("AKI prediction" OR "acute kidney injury") AND (surgery OR perioperative))

13. TITLE-ABS-KEY((chatbot OR chatgpt) AND (preoperative OR anesthesiology) AND evaluation)

14. TITLE-ABS-KEY((chatgpt OR chatbot) AND ("ASA score" OR "ASA status") AND "preoperative risk assessment")

15. TITLE-ABS-KEY((chatgpt OR chatbot) AND ("ASA score" OR "ASA status") AND "preoperative risk prediction")

16. TITLE-ABS-KEY((chatgpt OR chatbot) AND ("ASA score" OR "ASA status") AND prediction)

17. TITLE-ABS-KEY(("artificial intelligence" OR "large language model" OR "large language models" OR chatbot OR chatgpt) AND (perioperative OR surgery) AND ("clinical frailty index" OR "frailty index"))

18. TITLE-ABS-KEY(("artificial intelligence" OR "large language model" OR "large language models" OR chatbot OR chatgpt) AND (perioperative OR surgery) AND NSQIP)

19. TITLE-ABS-KEY(("artificial intelligence" OR "large language model" OR "large language models" OR chatbot OR chatgpt) AND (perioperative OR surgery) AND "AKI risk index")

20. TITLE-ABS-KEY(("artificial intelligence" OR "large language model" OR "large language models" OR chatbot OR chatgpt) AND (perioperative OR surgery) AND "Mini-Cog")

21. TITLE-ABS-KEY(("artificial intelligence" OR "large language model" OR "large language models" OR chatbot OR chatgpt) AND (perioperative OR surgery) AND SORT)

22. TITLE-ABS-KEY(("artificial intelligence" OR "large language model" OR "large language models" OR chatbot OR chatgpt) AND (perioperative OR surgery) AND "P-POSSUM")

23. TITLE-ABS-KEY(("artificial intelligence" OR "large language model" OR "large language models" OR chatbot OR chatgpt) AND (perioperative OR surgery) AND "EuroSCORE II")

Database: Cochrane Central Register of Controlled Trials (CENTRAL)

1. chatbot AND ("perioperative assessment" OR "preoperative evaluation" OR "anesthesia evaluation")

2. (artificial intelligence OR AI) AND chatbot AND (anesthesia OR anesthesiology)

3. "conversational agent" AND ("perioperative care" OR "preoperative screening" OR "anesthesia consultation")

4. ("virtual assistant" OR "digital assistant") AND (anesthesia OR perioperative)

5. "natural language processing" AND anesthesia AND (preoperative OR perioperative)

6. ("machine learning" OR "deep learning") AND chatbot AND ("anesthesia risk assessment" OR "preoperative evaluation")

7. "automated conversation" AND (anesthesia OR "perioperative care" OR "preoperative screening" OR "perioperative assessment")

8. "intelligent virtual agent" AND (anesthesiology OR "perioperative care" OR "preoperative assessment")

9. (artificial intelligence OR chatbot OR chatgpt) AND "revised cardiac risk index"

10. (artificial intelligence OR chatbot OR chatgpt) AND "frailty index" AND (surgery OR perioperative)

11. (artificial intelligence OR chatbot OR chatgpt) AND "revised cardiac risk index" AND (surgery OR perioperative)

12. (artificial intelligence OR chatbot OR chatgpt) AND ("AKI prediction" OR "acute kidney injury") AND (surgery OR perioperative)

13. (chatbot OR chatgpt) AND (preoperative OR anesthesiology) AND evaluation

14. (chatgpt OR chatbot) AND ("ASA score" OR "ASA status") AND "preoperative risk assessment"

15. (chatgpt OR chatbot) AND ("ASA score" OR "ASA status") AND "preoperative risk prediction"

16. (chatgpt OR chatbot) AND ("ASA score" OR "ASA status") AND prediction

17. ("artificial intelligence" OR "large language model" OR "large language models" OR chatbot OR chatgpt) AND (perioperative OR surgery) AND ("clinical frailty index" OR "frailty index")

18. ("artificial intelligence" OR "large language model" OR "large language models" OR chatbot OR chatgpt) AND (perioperative OR surgery) AND NSQIP

19. ("artificial intelligence" OR "large language model" OR "large language models" OR chatbot OR chatgpt) AND (perioperative OR surgery) AND "AKI risk index"

20. ("artificial intelligence" OR "large language model" OR "large language models" OR chatbot OR chatgpt) AND (perioperative OR surgery) AND "Mini-Cog"

21. ("artificial intelligence" OR "large language model" OR "large language models" OR chatbot OR chatgpt) AND (perioperative OR surgery) AND SORT

22. ("artificial intelligence" OR "large language model" OR "large language models" OR chatbot OR chatgpt) AND (perioperative OR surgery) AND "P-POSSUM"

23. ("artificial intelligence" OR "large language model" OR "large language models" OR chatbot OR chatgpt) AND (perioperative OR surgery) AND "EuroSCORE II"

Database: Google Scholar and OpenGrey / other grey literature sources

1. chatbot "perioperative assessment" OR "preoperative evaluation" OR "anesthesia evaluation"

2. "artificial intelligence" OR AI chatbot anesthesia OR anesthesiology

3. "conversational agent" "perioperative care" OR "preoperative screening" OR "anesthesia consultation"

4. "virtual assistant" OR "digital assistant" anesthesia OR perioperative

5. "natural language processing" anesthesia preoperative OR perioperative

6. "machine learning" OR "deep learning" chatbot "anesthesia risk assessment" OR "preoperative evaluation"

7. "automated conversation" anesthesia OR "perioperative care" OR "preoperative screening" OR "perioperative assessment"

8. "intelligent virtual agent" anesthesiology OR "perioperative care" OR "preoperative assessment"

9. chatbot OR chatgpt "revised cardiac risk index"

10. chatbot OR chatgpt "frailty index" surgery OR perioperative

11. chatbot OR chatgpt "revised cardiac risk index" surgery OR perioperative

12. chatbot OR chatgpt "AKI prediction" OR "acute kidney injury" surgery OR perioperative

13. chatbot OR chatgpt preoperative OR anesthesiology evaluation

14. chatgpt OR chatbot "ASA score" OR "ASA status" "preoperative risk assessment"

15. chatgpt OR chatbot "ASA score" OR "ASA status" "preoperative risk prediction"

16. chatgpt OR chatbot "ASA score" OR "ASA status" prediction

17. "artificial intelligence" OR "large language model" OR chatbot OR chatgpt perioperative OR surgery "clinical frailty index" OR "frailty index"

18. "artificial intelligence" OR "large language model" OR chatbot OR chatgpt perioperative OR surgery NSQIP

19. "artificial intelligence" OR "large language model" OR chatbot OR chatgpt perioperative OR surgery "AKI risk index"

20. "artificial intelligence" OR "large language model" OR chatbot OR chatgpt perioperative OR surgery "Mini-Cog"

21. "artificial intelligence" OR "large language model" OR chatbot OR chatgpt perioperative OR surgery SORT

22. "artificial intelligence" OR "large language model" OR chatbot OR chatgpt perioperative OR surgery "P-POSSUM"

23. "artificial intelligence" OR "large language model" OR chatbot OR chatgpt perioperative OR surgery "EuroSCORE II"
